# Breast Cancer Treatment in Integrated Care Process in Andalusia: The Challenge of Multidisciplinarity

**DOI:** 10.3390/ijerph191912728

**Published:** 2022-10-05

**Authors:** Carmen Rodríguez-Reinado, Ana Delgado-Parrilla, Juan Alguacil

**Affiliations:** 1 Clinical, Environmental and Social Transformation Epidemiology Research Group, Department of Sociology, Social Work and Public Health, University of Huelva, 21007 Huelva, Spain; 2 Centro de Investigación en Recursos Naturales, Salud y Medio Ambiente (RENSMA), University of Huelva, 21071 Huelva, Spain; 3 Consorcio de Investigación Biomédica en Red de Epidemiología y Salud Pública (CIBERESP), 28029 Madrid, Spain

**Keywords:** breast cancer, barrier, facilitator, multidisciplinary, treatment

## Abstract

Despite the increasing trend in the incidence of breast cancer in recent decades, mortality has decreased in developed countries. The general objective of the study is to analyse the functioning and organisation of the care process for breast cancer treatment in Andalusia (Spain) in order to identify possible barriers and facilitators that may be affecting its effectiveness and, therefore, the survival of the disease. A qualitative method was adopted based on 19 semi-structured interviews with health professionals from different specialities in two Andalusian provinces: Huelva (mortality rate higher than the national average) and Granada (mortality rate similar to the national average). Results show the existence of barriers (seasonal delays, low frequency of multidisciplinary meetings, lack of human and technical resources, difficulties in accessing treatment in certain populations, etc.) and facilitators (creation of multidisciplinary units and committees for breast pathology, standardisation of treatments, assignment of professionals with preferential attention to breast pathology, etc.) in the care process of breast cancer treatment. The combination of these barriers can have an impact on the accessibility, quality, and efficacy of the treatment, and in the long term, on survival from the disease.

## 1. Introduction

### 1.1. Epidemiologic Approach to Breast Cancer

Cancer is one of the main causes of morbidity and mortality worldwide [1]. According to estimates from the GLOBOCAN project [2], in 2020, approximately 19.3 million new cases of cancer were diagnosed in the world. At the international level, breast cancer (with 2,261,419 cases), is established as the most frequently diagnosed tumour, followed by cancers of the lung (2,206,771), colon and rectum (1,931,590), prostate (1,414,259) and stomach (1,089,103) [2]. In Spain, breast cancer represents the tumour with the third highest incidence in the general population (33,375) and the first in women [1].

Since the 1990s, in the countries of the global economic centre, the incidence of breast cancer has followed an increasing trend [3]. In contrast, mortality data reflect an opposite tendency [3,4,5,6]. This trend is reproduced in Spain with a decrease in breast cancer mortality from 1992 onwards [7].

### 1.2. Improvement in Survival Rates: Early Detection, Improvements in Treatment, and Greater Health System Efficiency

In the scientific literature, the decrease in mortality since the 1990s has been attributed to the combination of several factors: (1) early detection, (2) improvements in treatment, and (3) enhanced efficiency in health systems [6,7]. However, the specific weight that each factor has in improving the survival data of the disease is still unknown [4,8].

In relation to early detection and its impact on mortality rates, there is strong scientific evidence pointing to a greater probability of surviving breast cancer depending on the stage of the disease at the time of starting treatment [9,10]. Hence, early detection of breast cancer is one of the factors associated with increased survival [11]. Population screening programs are the most widespread and successful strategy for achieving an early diagnosis. Numerous investigations have attributed the decline in mortality from breast cancer in recent decades in Western countries to the implementation, and extension, of universal screening programs among the population aged 50 to 70 years [12,13,14].

Since the 1980s, improvements in treatments for the disease and the development of new therapies have been key factors in the decline in breast cancer mortality data [15]. The introduction of systemic therapies in the 1980s [16], for example, is associated with the decrease in mortality that occurred in the 1990s [15]. In the same line, the conclusions of a meta-analysis found a direct relationship between adjuvant therapy treatments and both the reduction in the risk of recurrence at 5 years and the annual rate of mortality from breast cancer at 15 years [17]. Likewise, a decrease in mortality is foreseen in the coming years [15] as a result of recent advances in therapeutic strategies based on the creation of more effective chemotherapy regimens and the development of adjuvant biological drugs [18].

Lastly, we found structural and organisational factors that may affect breast cancer survival [19,20,21,22]. Some studies show that in hospitals with a larger size and volume of patients, survival is greater [19,20,21,22,23]. A study carried out in Belgium, for example, showed that in larger hospitals with meetings of multidisciplinary teams, histological evaluation prior to surgery, breast-conserving surgeries, adjuvant radiotherapy after conservative surgery, and mammograms, with greater frequency, all these factors had a clear influence on better survival rates [23]. However, it is still unknown which factors related to hospital size have a greater impact on survival. 

### 1.3. The Breast Pathology Units: A Key Site for the Improvement of Treatment and Its Functional and Structural Organisation

Breast pathology requires a multidisciplinary approach to early detection, diagnosis, and treatment activities [24,25]. The tendency to develop increasingly individualised therapeutic strategies based on the characteristics of each patient translates into the need for specialists from different medical areas and different levels of care to intervene jointly in comprehensive patient care, with adequate coordination to achieve quality care [26]. In 1995, a report was published in the United Kingdom titled “A Policy Framework for Commissioning Cancer Services”, which recommended the creation of Breast Units made up of professionals specifically dedicated to breast pathology [27]. Three years later, in 1998, the first European Breast Cancer Conference (EBCC1) was held, highlighting the importance of promoting the creation of multidisciplinary teams dedicated to breast cancer [28].

In 2000, the European Society of Mastology (EUSOMA) published a document titled “Requirements for a unit specialised in breast cancer”, which became the starting point for working towards the goal of creating Breast Units in Europe [29]. This involves providing patients with the opportunity of a multidisciplinary approach to their disease, as compiled by the EBCC1 [28]. In 2003 and 2006, the European Parliament included the premises of this document in its recommendations. Thus, it recommends adopting a multidisciplinary approach to the management of breast cancer, given the complexity of the treatment of the disease and the need to coordinate multiple specialists [28,30,31,32].

According to EUSOMA, Breast Units are multidisciplinary care teams in which professionals from different specialities work, with exclusive or preferential dedication to breast pathology [29]. These units should carry out comprehensive and multidisciplinary management of breast cancer [31] and should be made up of specialists from different disciplines such as radiology, surgery, pathological anatomy, medical oncology, radiotherapy, and nursing, with dedication to and preferential training in senology [28,29].

In Spain, all the Autonomous Communities have established comprehensive plans and procedures for breast cancer patient care, embracing a comprehensive and multidisciplinary management approach with the necessary coordination of the different professionals involved [33]. In Andalusia, this approach was adopted in 2002 in the document titled “The Integrated Care Process for Breast Cancer (PAICM)”, published by the Ministry of Health. Drawing up this document, in which professionals from different fields participated, represented one of the first integrated care processes in the Andalusian public system [33]. Since its publication in 2002, two updates to the PAICM have been published [34]. In the latest version (published in 2011) [35], aspects such as the patient’s roadmap, information and safety for the patient, the skills of the different specialists involved, and management of patients with increased risk of breast cancer were included [33].

The extension in recent years of Multidisciplinary Units in Europe and Spain, then, is an established fact, with the consequent impact on the healthcare of breast cancer patients [24,25,36]. This multidisciplinary approach to breast cancer builds an overall vision of the disease, improving the outcomes of therapeutic strategies and responding to the expectations of the patients and professionals involved [33]. In this light, the relevance of this study is clear, as it aims to analyse the organisation and operation of the breast cancer treatment process to determine barriers and facilitators that may influence its effectiveness and, therefore, on survival rates for the disease.

## 2. Materials and Methods

### 2.1. Research Design and Ethical Approval

The method adopted in this study was qualitative. This type of methodology has proven useful for studying the factors and habits that affect health, as well as for planning and evaluating health services and policies [37]. This study was conducted following the ethical principles of the Declaration of Helsinki and the Belmont Report. The hospital ethics committee approved the study (PI 033/16). Before conducting the interviews, written informed consent was obtained from each participant. 

### 2.2. Study Settings

The scope of the study was the Autonomous Community of Andalusia, Spain. The unit of analysis was the care process in the treatment of breast cancer in the Andalusian public health system. The research was carried out in 4 hospitals (urban and rural) in 2 Andalusian cities: Granada and Huelva. These 2 cities were selected for their relevance to the object of study due to their epidemiological data on breast cancer mortality rates. Huelva is the Andalusian province that, with an incidence of breast cancer similar to the national average, presents the highest mortality data (23.74 × 10^−5^: 16.7 × 10^−5^) [38]. In contrast, Granada is the city with the lowest mortality but with an incidence of the disease similar to the Andalusian average.

### 2.3. Sample

The sampling was qualitative and theoretical [39]. The sample consisted of the population segment of health professionals working in breast cancer care in the Andalusian public health system. Thus, this segment of the population comprised the different professional profiles making up the Breast Pathology Unit (Table 1). The experience and discourses of these different profiles may be relevant to identifying factors in the organisation and operation of breast cancer care that may influence greater or better quality of care and, in consequence, mortality from the disease. 

#### 2.3.1. Sample Inclusion Criteria

1. Having at least 5 years of professional experience related to breast cancer care.
2. Having Spanish nationality.
3. Working in the public health system.

The total number of participants in the study was determined by applying the sampling saturation criterion [40].

#### 2.3.2. Recruitment of Participants

The hospitals were informed of the objectives and purpose of the research, and their participation in the study was requested. The different profiles of professionals were recruited through the directors of the different Breast Pathology Units. Table 1 below shows the distribution of participants according to their speciality.

### 2.4. Data Collection

Data collection was carried out between January 2019 and March 2021. The data collection technique was semi-structured individual interviews. Prior to carrying out the interviews, a topic script was designed according to the 4 dimensions on which the research objectives were based: (1) organisation of the integrated care process for breast cancer; (2) facilitators of and barriers to the effectiveness of breast cancer treatment; (3) evaluation of patient follow-up; and (4) coordination and material and human resources. Finally, a topic script was developed with a battery of 14 open questions (Table 2). Open items were chosen rather than closed ones in order to encourage participants to reflect on and discuss the basic dimensions of the analysis of the study. 

Before carrying out fieldwork, the relevance and understanding of the language used in the topic script were tested with 2 health professionals from the Andalusian public health system. After piloting the topic script, 19 semi-structured interviews were carried out. All individual interviews were conducted by 2 expert researchers. Before starting individual interviews, the participants were asked for their express consent to proceed with the audio recording of the session.

### 2.5. Analysis

The semi-structured individual interviews were all recorded in digital audio format. All recorded information was transcribed verbatim. A quality control analysis consisting of the random selection of different paragraphs for verification was conducted on the transcriptions. The Grounded Theory was applied as a method of analysis (Strauss, A., & Corbin, J. (1994) [39]. This methodological approach was adopted for its appropriateness to exploring this social health situation without explaining it on the basis of any preconceived theory analysis of the data was carried out. Therefore an inductive process was followed in data analysis [40,41]: (1) reading and re-reading the interview transcripts (open coding); (2) labelling the information according to the categories and codes (open coding and axial coding); (3) carrying out a relational analysis firstly of the categories and codes; and (4) describing the manifest and latent content of the categories and codes; (5) Subsequently the results were compared with data and theories from other studies.

All information was processed using qualitative data analysis software ATLAS.TIC version 8 (Scientific Software Development GmbH, Berlin, Germany) [42]. 

### 2.6. Rigor

Following Lincoln and Guba [43,44], Table 3 shows the strategies and techniques used to guarantee the scientific rigor of the study: 

## 3. Results

### 3.1. The Therapeutic Strategy after Breast Cancer Diagnosis: Breast Pathology Units and Comprehensive Case Management

Currently, in the public health system, the therapeutic treatment of breast cancer is carried out in the hospital setting by Breast Pathology Units [34]. The Units are responsible for the management, coordination, and execution of the care process from diagnosis and treatment to rehabilitation and follow-up for breast cancer patients [34].

The healthcare professionals interviewed positively valued the creation of Breast Pathology Units in their hospitals and, consequently, the units’ multidisciplinary approach to breast cancer treatment. In this regard, they highlighted the need for the units to have a functional organisation that ensured comprehensive management of the pathology in the patient due to the complexity of treatment for the disease:

*So it’s improved a lot, and the multidisciplinary management of breast cancer has improved a lot […]. The overall vision is much better.*

[RDHHU1]

*I create an overall vision of things, not a committee where there’s only the surgeon and the radiologist, and they tie everything up between them. I always think that the more people, the better.*

[MNGHU1]

Breast Pathology Units were created in hospitals after 2002 with the implementation of the Integrated Breast Cancer Care Process (hereafter PAICM) in the Andalusian public health system [34]. The healthcare professionals interviewed reported that the creation of the Units had not occurred homogeneously nor at the same time periods. Whether a particular hospital set up a unit or not depended on factors such as disease burden, economic resources, technical resources, human resources, etc.:

*The only thing that didn’t happen in unison was the process [… ]. The Infanta Elena (Huelva hospital) has only just begun to treat breast cancer. And this started happening in 2016.*

[RDHHU1]

*It was in 2006 when we set up the breast unit as it is here. The building work was done, and an ultrasound machine was bought because, later, the building work was extended with the merger, which brought in another ultrasound machine, and another mammographer.*

[RDHU1]

Consequently, during the fieldwork, differences were observed regarding the functioning and organisation of the Breast Pathology Units in the hospital settings studied. The first difference consisted of the clinical service of the hospital where the units are located. Thus, although in the majority of cases studied, the Breast Pathology Unit was located in the surgery area, in the case of urban hospital 1 in Granada, it was located in the gynecology department. According to the professionals interviewed, this meant that: 1) the leadership in the management and coordination of the Breast Pathology Unit corresponded to different medical specialities; 2) there were differences in the skills of the medical specialists responsible for breast cancer treatment. Thus, in urban hospital 1 in Granada, surgical interventions to treat breast cancer, among other medical interventions, were carried out by personnel specialised in gynecology, unlike the other hospitals analysed, where surgery was performed by surgeons. According to the healthcare personnel’s testimony, this difference was due to historical circumstances rather than questions of professional competence or scientific and clinical motivations:

*Why is gynaecology doing this and not surgery? Well, because it’s always been done that way […]. Traditionally we’ve done it, and we still do it ourselves. In other places, I know that only surgeons do it, and in other places, surgeons and gynaecologists. They all work just as well… many times for historical, traditional reasons rather than any other.*

[GGHU1]

In Spain, surgical treatment of breast pathology is included in the training and competencies of the speciality of general and digestive system surgery [45]. However, surgical treatment also forms part of the services and training portfolio of the speciality of obstetrics and gynaecology [45,46,47]. For this reason, there are a few hospitals in which breast pathology is shared between gynecology and surgery [46]. Interviewees reported that, prior to the implementation of the Andalusian Integrated Oncology Plan (2002), surgical and chemotherapy treatments in Granada Province were performed by the gynaecology service:

*A set of circumstances that made gynaecologists take charge of the breast cancer care process. I arrived in the 90s, and this was already underway. They were in charge of surgery and then systemic treatment.*

[RGHU2]

*Systemic cancer treatment was performed by gynaecologists, not oncologists. Then we radiation oncologists did the radiation treatment. […] since the mid-1980s […]. Not now.*

[RGHU1]

Since the implementation of the Andalusian Integrated Oncology Plan (2002), systemic treatment has become the responsibility of medical oncologists. However, interviewees reported that the incorporation of this medical speciality into the personnel of the Breast Pathology Unit of urban hospital 1 in Granada took place recently. This incorporation has not been free of tensions with gynaecologists:

*But I think that… I don’t know; we’re still at the beginning. So far, there’s still some friction between gynaecologists and medical oncologists, but anyway, in the end, we all get along.*

[RGHU1]

The medical professionals interviewed highlighted the trend towards more individualised therapeutic strategies, based on the characteristics of each patient, in breast cancer treatment. This requires the intervention of different specialists in the process of adapting the treatment. In this regard, participants reported that after confirming a positive case of breast cancer, and before beginning the clinical treatment of the disease, the Breast Pathology Unit proceeded to carry out a series of complementary or extension clinical tests to stage the tumour and personalise the treatment:

*When the cancer is confirmed, then what we do is staging through mammography and other complementary tests, resonances, etc., to decide which is the best treatment.*

[ORHHU1]

In most of the hospitals studied, the extension study was requested by the surgery service. Nevertheless, other specialities could also request extension tests if they deemed them necessary to adjust the treatment to clinical needs:

*There’s the breast pathology consultant, the surgeon, who performs the clinical examination of the patient, which is very important. He’s the one who asks for the complementary tests and the extension study and the one who gives the final diagnosis of the pathological anatomy that you have a malignant tumour in the breast.*

[ORGHU2]

The clinical tests in the extension study varied in each province analysed. In hospitals in the province of Huelva, computed axial tomography (CAT) of the abdomen and chest, bone gammagraphy and MRI, if appropriate due to age, clinical suspicion or breast density, were generally requested. In the province of Granada, the tests included bone grammagraphy, abdominal ultrasound and, if appropriate due to the severity of the case, a positron emission tomography (PET):

*The extension study for us is a bone gammagraphy to see the bones, an abdominal echography to see the liver, and, in some cases, depending on the initial extension of the disease we see, a PET scan is requested, a positron emission tomography.*

[MNGHU1]

*[…] women who have breast cancer are given a bone gammagraphy by protocol to check that they don’t have bone metastases, an extensive study of the abdomen and chest is done with the CT scan, in a word, to check things out… the basic extension study for all women.*

[ORHHU1]

#### The Tumour Committee: A Multidisciplinary Enclave

In general terms, the Multidisciplinary Committee was highly valued by the health professionals interviewed. In this regard, there was a consensus among them on the wisdom and relevance of adopting a multidisciplinary approach to the treatment of breast cancer. From their point of view, this provided personalised treatment for each patient, as well as better medical assistance. In addition, holding regular meetings with different medical specialities represented professional learning for them:

*I think the committees are a sure winner. I think there should be committees in all hospitals and for everything. Having a multidisciplinary vision of things is always positive. Also, you can learn. […] First, your personal learning and second, that the patients, in my opinion, are much better attended to when there’s an overall vision.*

[MNGHU1]

*I think it’s quite enriching because there are cases that are very simple, but also cases that lend themselves to discussion, and everyone participates. I think, for the patient, it’s much better when it’s not a single person making the decisions.*

[GGHU1]

To a large extent, this positive assessment of the Multidisciplinary Committee derived from participants’ perception of committees as a necessary and highly relevant instrument for complicated clinical cases of breast cancer. Thus, given the existence of such cases, they saw the meetings of the Multidisciplinary Committee as facilitating joint analysis of the clinical case by medical professionals from different specialities:

*When the case is clear, or it’s a small tumour, the surgeons operate on it from the start, but when there’s some alteration or something is a little out of the ordinary or raises doubts, then the action plan comes in… the action plan is agreed on.*

[OM2HHU1]

*I think that it works well and that it makes decisions that, sometimes more and sometimes less, attempt to improve overall care for women with breast cancer.*

[OM1HHU1]

Some interviewees highlighted as a strength that decision-making was shared regarding the type of treatment to prescribe in clinical cases that presented doubts. Thus, one participant perceived the committee as an administrative body that supported the clinical decision adopted for the patient, thereby exempting personnel from individual responsibility:

*It ensures that the treatment is the most suitable because two heads are better than one, and everything’s taken into account. I think that’s a strong point.*

[ORGHU1]

*I’m not the only one who decides what to do with that particular patient. That’s fine, especially when you’re not sure. The tiny nodule that goes for sentinel node surgery, no one has any doubts about it, but there are tumours that can be multifocal, that can be multicentric; it depends. The committee is an official body that supports the decision given for that particular woman.*

[GGHU1]

In the hospitals analysed, the main objective of the Multidisciplinary Committee was to study confirmed cases of breast cancer and establish the most appropriate therapeutic strategy for the patient. Some committees, however, went further than these main functions:

*First, the objective of the committee is to present newly diagnosed patients to make joint, multidisciplinary therapeutic decisions. Those are the basics, shall we say. And then other objectives are to establish common work protocols, to set up common research studies, and often also to comment on functional problems, delay problems, basic surgical issues, and specific speciality issues. Basically, what I’ve told you: care protocols, also to present some relevant study or bibliographical session on the subject. There are many things, but the basic one is to present the patients.*

[OM1HHU1]

The health professionals interviewed also positively valued the organisation and functioning of the Multidisciplinary Committees. However, there were differences between rural and urban hospitals. Generally, the Multidisciplinary Committees of the Breast Pathology Units were held on a weekly basis. In rural hospitals, however, a lower frequency was observed, with meetings only once a month. There was also less specialisation in the breast since there was a general Tumour Committee not specific to breast cancer. Both factors were perceived as weaknesses in the care process:

*The truth is that we work really, really well because we have weekly committees.*

[OMGHU2]

*[…] we have a really good relationship between professionals, and we’re very close, but I’d like, and this is something I’ve proposed to see if it could be done in the future, I’d like to meet at least once a week and for all the breast cancers diagnosed to be evaluated jointly by at least one oncologist, a surgeon and a radiologist, something that’s not being done at the moment. A tumour committee is held once a month for tumours in general […].*

[CHHR1]

Differences also emerged among the different hospitals regarding the work methodology of the Multidisciplinary Committees. Although, in general terms, the recently diagnosed and most problematic clinical cases were discussed in the committee, in urban hospital 1 in Granada, all tumours diagnosed, with the exception of benign tumours, were presented, along with every step taken in the therapeutic strategy for each breast tumour:

*There, the cases of most interest or that cause most doubts or that provoke debate as to whether one treatment or another is better are discussed. And the rest of the patients, well, through a consultation sheet or if not by phone or now with WhatsApp: "What about this patient?”.*

[ORHHU1]

*Another advantage that I’ve seen is that it’s a lot of work for the committee members, but all cases are discussed at all times, to decide on surgery, to decide on radiotherapy treatment, to decide on the best option if there’s a relapse.*

[ORGHU1]

Lastly, there were also differences in the composition of the multidisciplinary committees. In general terms, in the province of Huelva, there was less diversification in medical specialities among committee members (radiologists, pathologists, surgeons and medical oncologists and radiologists), compared to the provincial hospitals of Granada, with specialists in nuclear medicine, genetic counselling, psychology and plastic surgery.

### 3.2. Surgical Treatment of the Disease: Territorial Inequalities

The surgeons interviewed agreed in affirming that, in line with the findings of the recent scientific literature, more conservative and less invasive surgeries are currently performed than a few years ago, and argued for the effectiveness of the interventions:

*[…] in the past, when you had to operate on a patient, most of them underwent a mastectomy, axillary dissection and then chemotherapy; nowadays, this has changed a lot, fortunately.*

[CHHR1]

*In fact, when conservative surgery is indicated, it’s conservative surgery, and it has to be as conservative as possible.*

[MNGHU1]

Surgical strategies in the treatment of the pathology, once the patient has been diagnosed with breast cancer, can range from conservative surgery (lumpectomy or oncoplastic surgery techniques) to radical or simple mastectomy, with or without breast reconstruction [33]. The choice of one or another therapeutic approach depends on the size and location of the lesion, the size of the breast, the results of imaging tests, and patient preference [33].

Although the different options in the surgical treatment of the disease were standard in all Andalusian provinces, some differences were detected among the hospitals analysed. On the one hand, differences were observed regarding surgical treatment in relation to plastic surgery. Hospitals in Huelva had fewer resources for plastic surgery. Hence, sometimes, for certain techniques, the patient was referred to hospitals in other provinces. In contrast, hospitals in the province of Granada had plastic surgery tenders for breast reconstruction, as well as complex mammoplasty techniques, which allowed immediate breast reconstruction to be carried out in a single intervention:

*It could improve in terms of having more support, especially in plastic surgery. There are techniques that we have to refer to Seville as a reference centre. A lot of reconstructions, like abdominal grafts, aren’t done here; they’re done there.*

[CHHR1]

*We’re beginning to improve things because, for example, until now, we didn’t offer oncoplastic surgery, the topic of immediate reconstruction. We’d neglected that area a bit… call it neglected, call it eh…. Well, it wasn’t very clear how we were going to deal with it. One of the improvements that we are going to be making from 2019 is to work with the plastic surgery service for patients who could be candidates for immediate reconstruction and patients who are going to undergo mastectomy; it can be done.*

[GGHU1]

Some of the reasons that the surgical staff gave to justify the practice of breast reconstruction in a single operation were: (1) it does not change the mastectomy prognosis; (2) similar cosmetic results are obtained if the reconstruction is done in one or two stages; and (3) the single intervention reduces care pressure on the surgery service:

*Because the oncological results don’t vary. Because the cosmetic results practically don’t vary. And because of the pressure on care, the waiting list is greatly reduced, and it’s easier to operate on a patient once in a longer surgery than to have to operate on her twice. And the plastic surgery waiting lists are terrible. So for the patient whose breast we don’t reconstruct immediately, it’ll take a long time to reconstruct.*

[CGHU2]

Another difference was that in the Breast Pathology Unit in Granada hospitals, there was a tattoo consultation that aimed to improve patients’ self-image after undergoing a mastectomy and subsequent breast reconstruction:

*A few months ago, we opened a tattoo consultation in nursing. So the patient, even if she loses her areola or her nipple in the six months needed for the whole healing process, she’s then referred to tattoos.*

[CGHU2]

Regarding the waiting times for surgical treatment, in both provinces, the health personnel interviewed perceived the intervals between performing the diagnostic tests and the start of treatment as acceptable. In general, this interval was approximately 30 days from when the patient received the diagnosis until she underwent surgery:

*The waiting time that we have for breast cancer is 30 days. 30 days after the diagnosis, an intervention is performed.*

[CHHU1]

#### Chemotherapy and Radiotherapy Treatment: Improvements and Inequalities in Access

The health professionals interviewed reported that scientific advances in cancer treatments had contributed to the development of new drugs that expanded the possibilities of chemotherapy. They commented that the radiotherapy treatment had been simplified, reducing the number of sessions but maintaining effectiveness:

*At the level of chemotherapy, a lot more drugs have appeared, a lot more… A wider range of possibilities for women who perhaps were more limited by their molecular, hormonal or other profiles.*

*Four years ago, we used to do 25 sessions to treat a breast, and now we only do 15 because we’ve changed the divisions because new studies have come out that show that with the same efficacy, you can reduce women’s treatment by 10 sessions: “Wow! Well, that’s ten days less that they have to come for radiotherapy.*

[ORHHU1]

These advances in therapeutic strategies represented an improvement and facilitated the achievement of effective results with increasingly less invasive methods for patients. In addition, another advantage of advances in pharmacological treatment identified by respondents was that the number of visits to the health centre was reduced, thereby improving accessibility. However, some interviewees also indicated the existence of inequalities in access to conventional radiotherapy treatment in the province of Huelva, as it was only carried out in the provincial capital. Specifically, these accessibility barriers occurred in the case of patients with low socioeconomic status, residents in isolated rural municipalities -mainly in the mountains of the province- and those with insufficient public transport connections to the provincial capital:

*They’re normally poor people with few resources. Quite isolated towns in terms of public transport. For example, I have patients who come from towns where there’s one bus a day to Huelva. One, that’s it […]. And it’s three or two and a half hours walking from the town. […] You know? So that also limits you a lot when it comes to everything. […] There’s radiotherapy only in Huelva.*

[ORHHU1]

Likewise, the existence of inequalities in access to clinical trials of pharmacological treatments was revealed, depending on the area of patients’ residence. Thus, according to health professionals, clinical trials were usually carried out in provinces with larger populations and hospitals with greater resources. Hence, the province of Huelva had comparatively less access to clinical trials than the provinces of Granada or Seville:

*There are clinical trials, and we’d like to have more, obviously, when we think that a drug is going to benefit the patient. We’d like to have more. Sometimes among ourselves, if we think there’s a molecule that has good potential and it’s in Seville, we promote it. Patients are already asking us more and more about these possibilities.*

[CHHR1]

*At the moment, we have five or six clinical trials in breast cancer, which is good because we don’t have as many in other pathologies, but, for example, the Virgen del Rocio Hospital has 44 breast cancers, Macarena has, I think, 20-something.*

[OM1HHU1]

Regarding the organisation and functioning of the medical oncology service, in general terms, it was valued positively by the health personnel of the two provinces studied. According to those specialising in oncology, this service did not present long delays in waiting times for treatment. The interval from the time of surgery to the start of chemotherapy was approximately 4 to 6 weeks in all the hospitals analysed. However, if the case was urgent, interviewees from both provinces stated that treatment could begin the following day:

*Well, the service is very well organised. In other words, it’s really organised, and there are no delays […] the first visit that medical oncology does ranges between a week and 10 days.*

[OMHHU1]

*Well, we, I do chemotherapy if I see the lady and I want to give her chemotherapy that day or the next day, if it’s urgent, it’s urgent. […] obviously, if I can schedule it a little, I do schedule it, but if I need it to be tomorrow, it’s tomorrow. [… ]*

[OMGHU2]

Similarly, the time intervals that elapsed until radiotherapy treatment started were positively valued. In both provinces analysed, radiotherapy treatment was scheduled for around four weeks after surgery, the minimum time for ensuring that surgical wounds have healed. These time intervals were seen as positive by health personnel specialised in radiotherapy:

*Normally we don’t start radiotherapy until at least a month has passed since the surgery because the scars are open, and if we radiate a breast that hasn’t closed properly yet, the wound can open, and you can make a bloody mess of it.*

[ORHHU1]

*Yes, I’m talking about my service, radiation oncology. Right now, at the moment, we’re up to date. Every patient who gets presented to the committee, we see them either that week or the next.*

[ORGHU2]

### 3.3. Human, Technical and Economic Resources in the Treatment of Breast Cancer: Seasonal Delays

The hiring of personnel with a preferential dedication to breast tumours constitutes a quality criterion in accordance with the recommendations of the European Society of Breast Cancer Specialists [29]. In this respect, differences were observed between urban and rural hospitals. In urban hospitals, there were surgeons with preferential care for breast pathology (Huelva: four surgeons; Granada one: from 8–10 gynaecologists; Granada: five surgeons), unlike the rural hospital where there were none. 

The health professionals of both provinces reported that the time intervals were in accordance with institutionally established protocols. However, they also indicated that the different services -radiology, surgery, oncology, etc.- usually presented seasonal delays during holiday periods since healthcare staff were reduced and no personnel were contracted for their replacement:

*There are periods that are more difficult. Vacations, vacations are more difficult because there’s less staff in all areas, in all services, so what normally can take 6 weeks, maybe takes 8.*

[OMGHU2]

*On average, Christmas is coming now, and there’s no place to put all of them, so they have to wait till the next week.*

[GGHU1]

Beyond these occasional delays, the specialised health personnel of the two Andalusian provinces studied perceived that the provision of human resources was insufficient and affirmed that, if the work proceeded properly and without undue delay, it was due to the commitment and extra work of the health staff:

*I believe that health should be something fundamental on the political level and shouldn’t be a subject for change for elections and a political football, now yes and now no, and now… So, from my point of view, the professionals who work, we get 100% involved in trying to make this go as well as possible, but the limitations are always from the hospital up. That there are no resources, there’s no money, and I won’t hire you anymore… I think that in radiotherapy, we’re working with three fewer technicians.*

[ORHHU1]

Hence, one of the main barriers detected by specialised health staff was the lack of human resources needed to carry out adequate comprehensive management of breast pathology:

*I don’t know if there’d be a possibility of… I think the manager’s doing it, looking for more activity in the afternoon, there’s an emergency plan, but sometimes anaesthesiologists are also lacking.*

[OMHHU1]

*Health here in Andalusia is quite limited, but even so, we manage to meet the deadlines required of us.*

[ORHHU1]

Rural hospitals had specific characteristics. For one, the staff of the rural hospital in the province of Huelva reported a structural human resources problem. They explained that the hospital workforce was unstable, with frequent turnover in healthcare staff. For this reason, they identified the need to implement a loyalty plan so that residents coming to the hospital to train and accumulate professional experience would not decide to leave for another hospital once this stage was over:

*The problem of small hospitals is always human resources. […] I see that most people who’ve just arrived come to one of these hospitals, earn points, gain experience, and when […] they’re already at the end of their training, they leave […] We‘ve lost two radiologists who left for the Macarena recently. Almost half of the staff of the surgery service had to be replaced. That’s the criticism I make of small hospitals, which is that management has to do something to retain staff because if staff isn’t retained, they have a quick turnover, and the quick turnover ultimately ends up resulting in a gradual deterioration of quality.*

[CHHR1]

Likewise, the health staff of the two provinces analysed indicated the need to expand technical resources, fundamentally to increase the number of operating theatres. Resources were also called for to improve extension studies for the appropriacy and individualisation of treatment. In the case of the urban hospital in Huelva, one specific demand was to improve MRI techniques since the existing deficiency had the effect of lengthening diagnostic times:

*More operating theatres, exactly, so that surgeons have more resources.*

[OMHHU1]

*And the staff’s not only required for basic field care, which is what I say, but we also need resources to carry out the whole preliminary study, all the preliminary protocols and everything that needs to be done before starting up a test. Because the test isn’t done, and that’s it. It takes prior preparation that takes a long time. So, of course, we need resources.*

[ORGHU2]

*The MRI often takes a month and delays the definitive diagnosis because the MRI should be shortened, we need more facilities for MRI, and that’s pending. I think that ultimately it’s resources, radiology resources and MRI resources to improve those times.*

[ORHHU1]

## 4. Discussion

The process of treating breast cancer has evolved substantially in the last decade, in both the clinical and organisational dimensions, achieving improved results in survival rates [6,7,15,17]. Although the influence of the organisational aspects of breast cancer treatment on survival from the disease is known [48], there is still little scientific production in this field that enables us to deepen our knowledge and establish classifications concerning the weight of each factor in incidence.

One of the main advances in the organisation of breast cancer care in Europe in the last decade has been the creation of Multidisciplinary Units [49]. In this regard, studies have concluded that the management of cancer patients through Multidisciplinary Units not only changes and improves treatment but also significantly increases survival [50,51,52,53]. In our research, all the hospitals studied had set up Multidisciplinary Units for breast cancer care. This factor was highlighted by health professionals as one of the main strengths of the Integrated Care Process for Breast Cancer implemented by the Andalusian Government. However, despite the importance attributed to it, no hospital had carried out quality accreditation for Multidisciplinary Units. In this regard, the positive impact of carrying out a review/audit procedure and establishing an accreditation system for the units is known not only for guaranteeing quality care but also for standardising services, which constitutes one of the factors that reduce breast cancer mortality [48].

In Spain, the implementation, evolution, and development of Multidisciplinary Units for breast cancer treatment have not been homogeneous [33,54]. This heterogeneity is reflected in differences found among the different hospitals studied in the organisation of the care process for breast cancer. These findings allow us to affirm the existence of organisational and operational differences in multidisciplinary units stemming from the size of the hospital and its geographical location. Other studies have found similar results, establishing a correlation between larger hospitals and better organisation of the units and, therefore, better survival rates [19,20,21,22,23]. Our research revealed a lack of economic, human and technical resources in smaller and rural hospitals, representing a barrier to the accessibility and effectiveness of breast cancer treatment.

The main organisational differences found in the Breast Pathology Units of the different hospitals were: (1) the clinical expertise in which they were located; (2) the medical expertise performing breast surgery; (3) the composition and frequency of the Multidisciplinary Committee (composition and frequency); (4) the therapeutic options available (surgery and chemotherapy); (5) economic and human resources. In this regard, research has shown that a decrease in variations in medical practice in breast cancer care improves survival [55]. Therefore, the urgent need to unify and standardise the care processes of the Multidisciplinary Units is evident.

**Figure 1:**
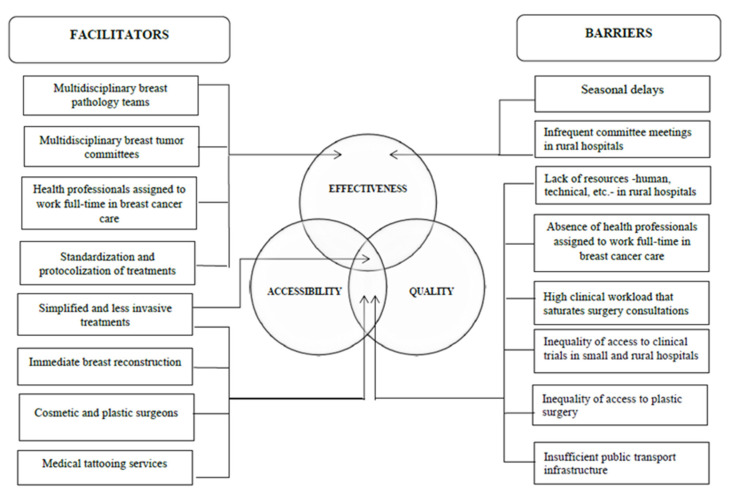
Barriers and facilitators in breast cancer treatment.

These organisational differences shape the facilitators and barriers in the use of the care process for breast cancer, specifically in the accessibility and efficacy of the treatment, as reflected in Figure 1 below:

The barriers identified in the study have been highlighted in other investigations as factors that can influence survival from the disease: delays in care [56,57,58]; accessibility of hospitals and surgical and oncological treatments [59,60]; frequency of multidisciplinary meetings [23]; adequate availability of health professionals with preferential or exclusive attention to breast pathology [31,61,62]; and, in general, allocation of economic resources to the health system [63].

In this regard, it is worth mentioning that Andalusia was the Spanish region that presented the highest cancer mortality rates in 2021 compared to all other Spanish regions [64]. In addition, it is the region with the third highest poverty rate in Spain [65].

### Study Limitations

The methodology used in the study does not allow clear associations with or influences on differences in the survival data of the provinces analysed to be identified.

## 5. Conclusions

A series of barriers with an impact on treatment accessibility, efficacy and quality were identified in breast cancer care. Thus, the hypothesis was deduced that these barriers might be influencing survival from the disease. Therefore, the relevance of and need for quantitative studies that can measure the impact of these barriers on survival rates is suggested. 

## Figures and Tables

**Table 1:** Professional profiles recruited for the study.

Speciality Area	N	Age	Sex	Province	Professional Background (Years)	Professional Background with Preferential Dedication to Breast Pathology (Years)
**Surgeons**	3	60	Man	Granada	29	12
		39	Man	Huelva	10	--
		55	Woman	Huelva	28	15
**Oncology**	4	34	Woman	Granada	9	4
		48	Woman	Granada	16	12
		32	Man	Huelva	8	7
		58	Man	Huelva	30	20
**Diagnostic Radiology**	5	55	Woman	Granada	13	9
		47	Man	Granada	28	15
		50	Man	Huelva	20	18
		62	Man	Huelva	24	11
		55	Woman	Huelva	24	5
**Radiotherapy**	3	33	Woman	Granada	6	2
		64	Woman	Granada	28	12
		35	Woman	Huelva	11	7
**Pathological Anatomy**	2	44	Woman	Granada	18	6
		47	Woman	Huelva	20	--
**Gynecology**	2	43	Woman	Granada	17	4
		65	Man	Granada	36	17
**Nuclear Medicine**	1	44	Woman	Granada	9	9
**Total**	19					

**Table 2:** Topic script.

**1**	Can you describe the integrated care process for breast cancer in Andalusia? What are the strengths and barriers that exist?
**2**	In your opinion, how is the care process for breast cancer treatment in your hospital? What are the strengths and barriers that exist?
**3**	Specifically, how is your service working in relation to breast cancer treatment care? Is it effective?
**4**	Is there a multidisciplinary approach to breast treatment in your hospital? How is it achieved? What are the strengths and barriers that exist?
**5**	How is the coordination between the service with the rest of the services and professionals involved in the diagnosis, treatment, and follow-up of breast cancer patients?
**6**	Regarding human resources, how many professionals in your speciality with preferential attention to breast pathology does the hospital have? Do you consider them sufficient? Do you think there are aspects to improve?
**7**	How do you rate the financial resources of the hospital dedicated to breast pathology? Do you consider them sufficient? Do you think there are aspects to improve?
**8**	What decisions would you make to improve patient care in your hospital?

**Table 3:** Scientific Rigor Criteria.

Criteria	Techniques Performed and Application Procedures
**1. Credibility**	
1.1. Prolonged Engagement	Fieldwork between 2019 and 2021.
1.2. Persistent observation	Circular process of data collection and analysis.
1.3. Triangulation of data	Researchers from different scientific disciplines contrasted the coding performed and the analysis of the information.
1.4. Peer debriefing	Exposition of the research design (sample, recruitment strategies, and information collection instruments) to two researchers.
**2. Transferability**	
2.1. Thick Descriptive Data	A detailed description of the health context (integrated care process for breast cancer), study sites (rural and urban hospitals), and participants’ profiles.
**3. Dependability and Confirmability**	The report was evaluated by an external researcher and health professional in breast cancer in the hospital.

## Data Availability

The data presented in this study are available on request from the corresponding authors. The data are not publicly available due to ethical.
